# Assisted Phytoremediation between Biochar and *Crotalaria pumila* to Phytostabilize Heavy Metals in Mine Tailings

**DOI:** 10.3390/plants13172516

**Published:** 2024-09-07

**Authors:** Marcos Rosas-Ramírez, Efraín Tovar-Sánchez, Alexis Rodríguez-Solís, Karen Flores-Trujillo, María Luisa Castrejón-Godínez, Patricia Mussali-Galante

**Affiliations:** 1 Doctorado en Ciencias Naturales, Universidad Autónoma del Estado de Morelos, Av. Universidad No. 1001, Col. Chamilpa, Cuernavaca 62209, Morelos, Mexico; marcos.rosas@uaem.edu.mx (M.R.-R.); anakaren.florestru@uaem.edu.mx (K.F.-T.); 2 Laboratorio de Investigaciones Ambientales, Centro de Investigación en Biotecnología, Universidad Autónoma del Estado de Morelos, Av. Universidad No. 1001, Col. Chamilpa, Cuernavaca 62209, Morelos, Mexico; alexis.rodriguez@uaem.mx; 3 Centro de Investigación en Biodiversidad y Conservación, Universidad Autónoma del Estado de Morelos, Av. Universidad No. 1001, Col. Chamilpa, Cuernavaca 62209, Morelos, Mexico; 4 Facultad de Ciencias Biológicas, Universidad Autónoma del Estado de Morelos, Av. Universidad No. 1001, Col. Chamilpa, Cuernavaca 62209, Morelos, Mexico; mlcastrejon@uaem.mx

**Keywords:** biochar, mine tailing, heavy metals, phytostabilization, *Crotalaria pumila*, hyperaccumulator

## Abstract

The increasing demand for mineral resources has generated mine tailings with heavy metals (HM) that negatively impact human and ecosystem health. Therefore, it is necessary to implement strategies that promote the immobilization or elimination of HM, like phytoremediation. However, the toxic effect of metals may affect plant establishment, growth, and fitness, reducing phytoremediation efficiency. Therefore, adding organic amendments to mine tailings, such as biochar, can favor the establishment of plants, reducing the bioavailability of HM and its subsequent incorporation into the food chain. Here, we evaluated HM bioaccumulation, biomass, morphological characters, chlorophyll content, and genotoxic damage in the herbaceous *Crotalaria pumila* to assess its potential for phytostabilization of HM in mine tailings. The study was carried out for 100 days on plants developed under greenhouse conditions under two treatments (tailing substrate and 75% tailing/25% coconut fiber biochar substrate); every 25 days, 12 plants were selected per treatment. *C. pumila* registered the following bioaccumulation patterns: Pb > Zn > Cu > Cd in root and in leaf tissues. Furthermore, the results showed that individuals that grew on mine tailing substrate bioaccumulated many times more metals (Zn: 2.1, Cu: 1.8, Cd: 5.0, Pb: 3.0) and showed higher genetic damage levels (1.5 times higher) compared to individuals grown on mine tailing substrate with biochar. In contrast, individuals grown on mine tailing substrate with biochar documented higher chlorophyll *a* and *b* content (1.1 times more, for both), as well as higher biomass (1.5 times more). Therefore, adding coconut fiber biochar to mine tailing has a positive effect on the establishment and development of *C. pumila* individuals with the potential to phytoextract and phytostabilize HM from polluted soils. Our results suggest that the binomial hyperaccumulator plant in combination with this particular biochar is an excellent system to phytostabilize soils contaminated with HM.

## 1. Introduction

Mine tailings are naturally multi-elemental residues, containing hazardous elements like heavy metals (HM) [1]. When HM are bioavailable, they bioaccumulate in organisms, resulting in biomagnification through the food chain, which puts humans and environmental health at risk [2,3].

The contamination resulting from mine tailings does not only cause an increase in HM concentration on a fine spatial scale, but also it can be extended to places kilometers away, due to erosion and leaching. Polluted soils by HM can limit the establishment of plant species, because they bioaccumulate HM in their tissues, restricting growth and causing morphological, physiological, and genetic alterations. In addition, if HMs are biomagnified, then the distribution and abundance of populations, the community structure, and the ecosystem dynamics [2,4] may be modified through the trophic chain. For these reasons, the remediation of soils contaminated by HM becomes necessary. 

In this sense, bioremediation is a commonly used technique, particularly phytoremediation, which has several advantages regarding conventional physicochemical processes, such as low cost, local application, which can result in the generation of a plant cover, thereby reducing erosion and leaching from the soil, as well as generating organic material, which reduces the bioavailability of HM [4,5].

Phytoremediation uses different plant species, including herbaceous plants, shrubs, and trees. These usually grow on sites contaminated with HM and help to avoid soil erosion, limiting the dispersion of contaminants. They have a short life cycle, wide geographic distribution [6,7], and tend to be native species to the contaminated site, preserving local biodiversity. These species have developed adaptive and survival strategies adequate for existence in adverse circumstances, including acid soil conditions and compacted substrates with low availability of nutrients and water [8]. Specifically, phytoextraction implies the absorption of HM from the ground and their translocation into the aerial parts of the plants, for example, the leaves, that later are collected and removed from the contaminated area [9]. Phytostabilization involves the establishment of a plant cover and organic amendments to immobilize HM through the absorption and accumulation in the root system, thus decreasing contamination levels in the affected site [10].

Due to the lack of resources and unfavorable conditions that mine tailings represent for plant life, organic amendments are used to help them grow more easily; a process known as assisted phytostabilization [11]. Some organic amendments can immobilize HM, forming highly insoluble compounds or absorbing them in such a way that they are not bioavailable for plants [12]. Over the last few years, great interest has been shown in the use of plant species in combination with organic amendments for polluted soils with HM with the aim of reducing the bioavailability of HM [13], thus lowering the risk of environmental exposure to these toxic elements [14,15].

Biochar is a material rich in carbon, with a large specific surface area, abundant pore structure, and different surface functional groups that have demonstrated a positive impact on the physical, chemical, and biological properties of the soil [16]. In this study, we propose using the waste material from coconuts as raw material for making biochar. Coconut palms are a perennial crop common to coasts and tropical regions, placing Mexico as one of the ten major producers of coconuts at a global scale [17]. Close to 25% are converted into biomass using coconut shells. However, the production of this material is not organized well since a lot is directly disposed of, left where it can cause illness, or else burned, which contaminates the environment, contributing to global warming [18]. Hence, it is important to offer an alternative for the correct management of coconut shells and how to turn them into biochar. 

To determine if a plant has the potential to be used in phytoremediation processes, it is important to analyze the effects derived from HM bioaccumulation. This can be evaluated through different biomarkers, such as morphological (biomass), physiological (chlorophyll content), and genotoxic damage (comet assay: a highly sensitive, simple, and fast technique for the analysis of the DNA damage in individual cells [19], which gives information about the overall plant health after exposure to genotoxins such as HM [2,20]).

*Crotalaria pumila* Ort (Fabaceae) can bioaccumulate HM, such as Cd, Cu, Fe, Pb, and Zn, in roots and leaves [21], while being able to grow naturally in HM-contaminated sites. For this reason, it is considered a pioneering species [22]. *C. pumila* has wide geographic distribution, high germination percentages, produces biomass, and is native to areas contaminated by HM. As for coconut fiber, it has been documented that it improves soil and has the capacity to immobilize HM. For this reason, in this study, we document the potential of biochar made with coconut fiber mixed with mine tailings rich in HM in order to evaluate: (1) the capacity of *C. pumila* to bioaccumulate and translocate HM; (2) the effects of bioaccumulation of HM on the morphological, physiological, and genetic damage of *C. pumila* individuals growing on mine tailing substrate, with and without biochar. It is hoped that the combination of *C*. *Pumila*-biochar, made with coconut fiber, phytostabilizes HM contained in the mine tailing substrate, thereby reducing the transference of HM to the aerial parts of the *C. pumila* individuals and limiting HM entering the food chain.

## 2. Results

### 2.1. Physicochemical Characterization and Heavy Metal Analysis of the Coconut Fiber Biochar

According to the physicochemical characterization, the biochar showed a pH value of 10, 4.4% of O.M., 10.5 mg kg^−1^ of phosphorous, 0.033 mg kg^−1^ of nitrogen, 2.6 mg kg^−1^ of carbon, and a C/N ratio of 78. According to the reference values established in the Mexican standard, NOM-021-SEMARNAT-2000, the coconut fiber biochar is a strongly alkaline substrate with a high O.M. proportion. In contrast, concerning the C/N ratio and phosphorous content, the observed values were situated in the medium range. Neither metal was detected, except for Zn (35 ± 5 mg kg^−1^).

### 2.2. Bioaccumulation of Heavy Metals in Root and Leaf Tissue in C. pumila Individuals That Grow in Mine Tailing Substrate and Mine Tailing Substrate/Biochar

**Figure 1:**
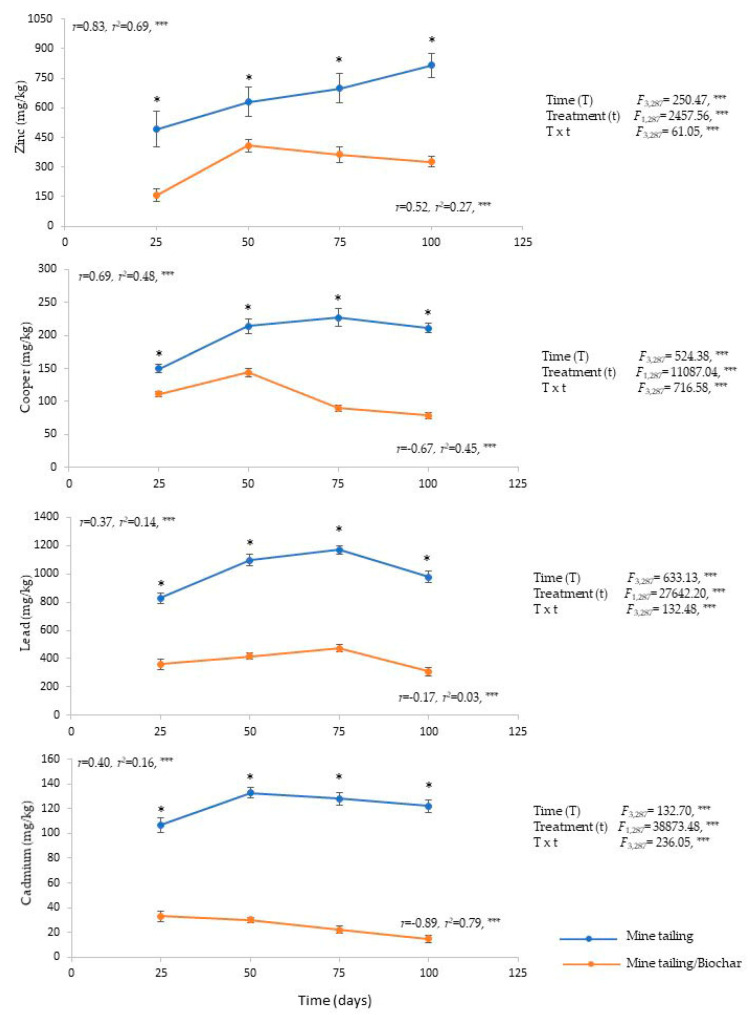
Heavy metal concentration (average ± standard error), two-way ANOVA to determine the effect of treatment, time, and interaction (treatment × time) in root of *C. pumila* growing under greenhouse conditions. Regression analysis between exposure time and heavy metal concentration in root. The asterisks denote significant differences between treatments by exposure time with *p* < 0.05 (Tukey). ANOVA test: *** = *p* < 0.001, * = *p* < 0.05.

Root: *C. pumila* individuals bioaccumulated Cd, Cu, Pb y Zn in their roots. The results of a two-way ANOVA show that the time, treatment, and interaction time × treatment, had a significant effect on the *C. pumila* bioaccumulation of these four metals (Figure 1). In general, the individuals growing on the mine tailing/biochar substrate registered a significantly lower concentration of Cd, Cu, Pb y Zn in their roots (Tukey test) in comparison with individuals growing on the mine tailing substrate, independent of the time of exposure (Figure 1).

Regression analysis showed a positive and significant relationship between exposure time to the mine tailing substrate and the bioaccumulation of 100% of the HM. In other words, the longer the exposure time, the greater the level of bioaccumulation achieved in *C. pumila* individuals. In the treatment with mine tailing substrate and biochar, a positive and significant relationship was recorded only for Zn. In contrast, for the remaining metals (Cd, Cu, and Pb), the relationship was significant but negative. In other words, the longer the exposure time, less bioaccumulation was achieved by *C. pumila* individuals (Figure 1).

The individuals growing on the mine tailing substrate presented the following average pattern of bioaccumulation (mg kg^−1^) of metals in roots: Pb (1027.25) > Zn (659.25) > Cu (200.50) > Cd (122.50), while for the individuals growing on the mine tailing/biochar substrate, the average pattern for bioaccumulation was: Pb (389.75) > Zn (313.75) > Cu (106.25) > Cd (25).

We detected the following pattern of metal immobilization using coconut fiber biocarbon added to mine tailings, using *C. pumila* as a model: Cd (79.7%) > Pb (61.6%) > Zn (53.3%) > Cu (47.3%).

**Figure 2:**
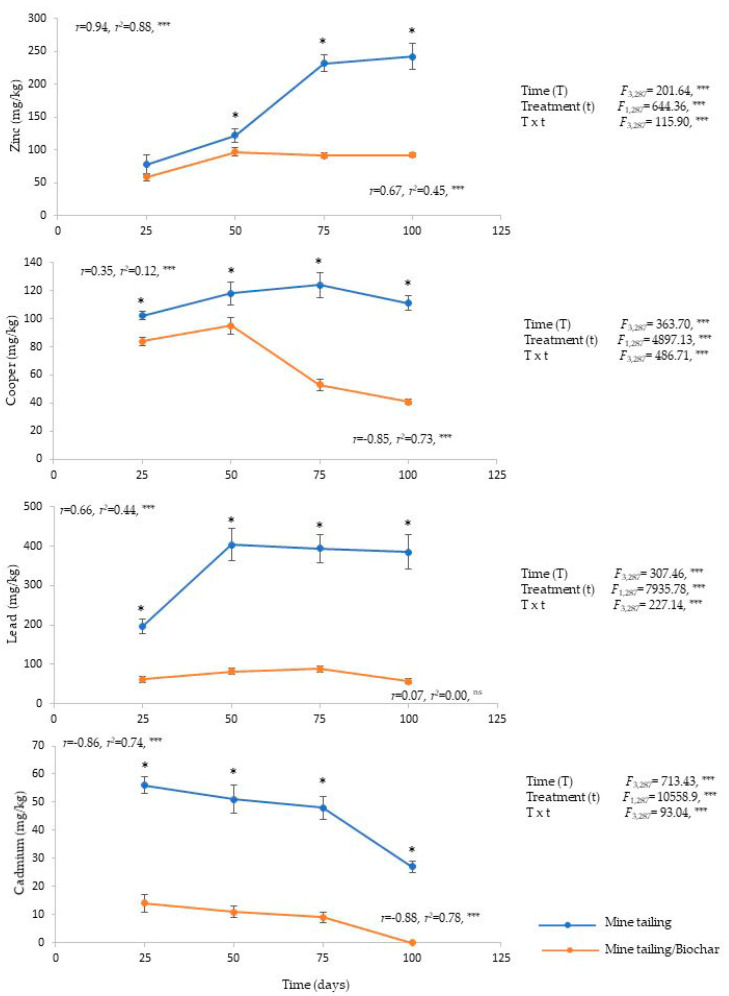
Heavy metal concentration (average ± standard error), two-way ANOVA to determine the effect of treatment, time, and interaction (treatment × time) in leaves of *C. pumila* growing under greenhouse conditions. Regression analysis between exposure time and heavy metal concentration in root. The asterisks denote significant differences between treatments by exposure time with *p* < 0.05 (Tukey). ANOVA test: *** = *p* < 0.001, * = *p* < 0.05. n.s. = not significant differences.

Leaf tissue: Cd, Cu, Pb, and Zn were bioaccumulated in the leaf tissue of *C. pumila* individuals. The results showed a significant effect of time, treatment, and time × treatment interaction on the bioaccumulation of these elements (Figure 2). In general, we found significant differences between treatments in all exposure times, with individuals growing on the mine tailing/biochar substrate registering lower levels of HM bioaccumulation compared to individuals growing on mine tailing substrates (Figure 2).

In addition, for the individuals growing on the mine tailing substrate, a positive and significant relationship was found between the exposure time and the bioaccumulation of all HM, except for Cd, which presented a significant but negative relationship.

The individuals that grew on the mine tailing substrate presented the following average pattern of bioaccumulation (mg kg^−1^) of HM: Pb (345.25) > Zn (168.75) > Cu (113.75) > Cd (45.50), while the individuals grown on the mine tailing/biochar substrate registered the following pattern: Zn (84.50) > Pb (72.25) > Cu (68.25) > Cd (8.5).

### 2.3. Metal Bioconcentration and Translocation Factors in Roots and Leaves in C. pumila Individuals Growing on Mine Tailing and Mine Tailing/Biochar Substrates

The bioconcentration factor (BCF) registered the following pattern Cd > Cu > Zn > Pb in individuals growing on mine tailing substrates, in both structures (roots and leaves). In contrast, the BCF in individuals that grew on the mine tailing/biochar substrate presented the following pattern Cu > Cd > Zn > Pb in both structures. In general, the BCF values were significantly lower in individuals growing on the mine tailing/biochar substrate than those growing on the mine tailing substrate (Table 1).

Meanwhile, the translocation factor (TF) was less than one for both treatments, presenting the following pattern: Cu > Cd > Pb > Zn. The individuals grown on mine tailing/biochar substrate presented significantly less TF of non-essential metals (Cd and Pb) compared to the individuals that were grown on the mine tailing substrate. In contrast, the individuals grown on the mine tailing substrate presented significantly less TF of essential metals (Cu and Zn) compared to the individuals gown on the mine tailing/biochar substrate (Table 1).

According to the Lam’s model, *C. pumila* could be considered an hyperaccumulator of Pb and Cu and accumulator of Cd and Zn in roots. On the other hand, *C. pumila* could be considered an hyperaccumulator of Pb and an accumulator of Cu, Cd and Zn in leaves (Table 2).

### 2.4. Effect of the Substrate (Mine Tailing and Mine Tailing/Biochar) on C. pumila Biomass

**Figure 3:**
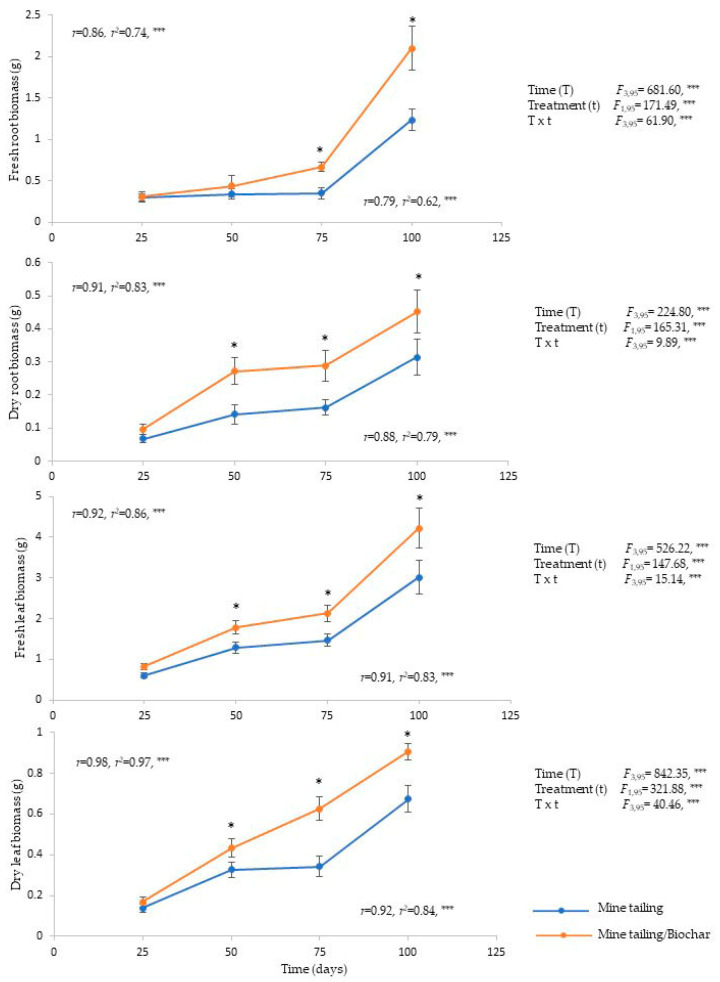
Average (±standard error) biomass of *C. pumila* roots and leaves growing in greenhouse conditions on mine tailing substrate and mine-tailing/substrate. Two-way ANOVA to evaluate the effect of time (100 days) and treatment on root and leaves biomass characters, and simple regression analysis to evaluate the relationship between exposure time to the substrate and biomass characters. The asterisks denote significant differences between treatments by exposure time with *p* < 0.05 (Tukey). ANOVA test: *** = *p* < 0.001, * = *p* < 0.05.

The results of the present study showed a significant effect of time, treatment, and time × treatment interaction on the biomass of *C. pumila*, fresh root biomass, dry root biomass, fresh leaf biomass, and dry leaf biomass (Figure 3). In general, from day 50 of exposure, *C. pumila* individuals growing on the substrate mine tailing/biochar registered significantly greater biomass compared to the individuals growing on the mine tailing substrate. The regression analyses showed a positive and significant relationship between the exposure time to the substrate and 100% of the biomass traits analyzed. In other words, the longer the exposure time, the greater biomass was registered in *C. pumila* individuals (Figure 3).

### 2.5. Effect of the Substrate (Mine Tailing and Mine Tailing/Biochar) on Chlorophyll a and b Content in C. pumila Individuals

**Figure 4:**
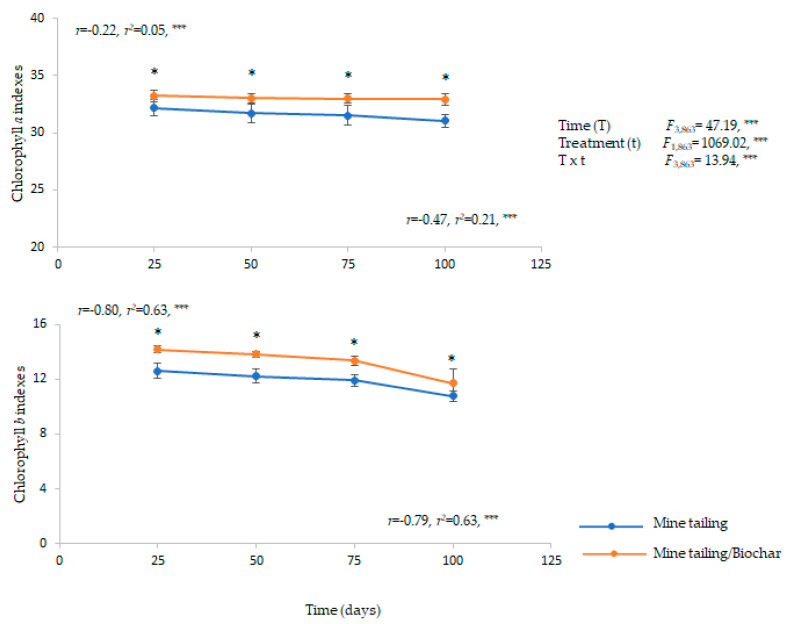
Average ± standard error of chlorophyll *a* and *b* from leaves from *C. pumila* growing in greenhouse conditions on mine tailing substrate and mine-tailing/substrate. Two-way ANOVA to evaluate the effect of time (100 days) and treatment on chlorophyll content from leaves, and simple regressions analysis to evaluate the relationship between exposure time to the substrate and chlorophyll content. The asterisks denote significant differences between treatments by exposure time with *p* < 0.05 (Tukey). ANOVA test: *** = *p* < 0.001, * = *p* < 0.05.

The results showed a significant effect of time, treatment, and time×treatment interaction on chlorophyll *a* and *b* in *C. pumila* individuals (Figure 4).

It was found that *C. pumila* individuals presented a decrease in chlorophyll *a* and *b* concentration across time, presenting significant differences between treatments in all exposure times, while those individuals grown on mine tailing with biochar showed significantly higher concentration of chlorophyll *a* and *b*, compared to individuals grown on mine tailing (Figure 4).

Furthermore, the regression analysis showed a significant and negative relationship between the exposure time and chlorophyll *a* and *b* concentration in both treatments, as the exposure time increases, the chlorophyll *a* and *b* concentration decreases in *C. pumila* individuals (Figure 4).

### 2.6. Effect of the Substrate (Mine Tailing and Mine Tailing/Biochar) on Genetic Damage in C. pumila Individuals

**Figure 5:**
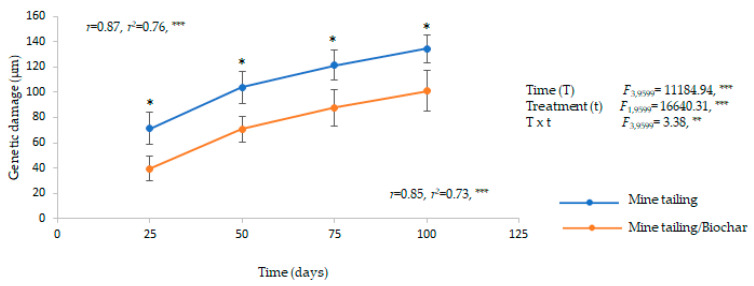
Average ± standard deviation of genetic damage (single strand breaks) in foliar tissue from *C. pumila* growing in greenhouse conditions on mine tailing substrate and mine tailing/substrate. Two-way ANOVA to evaluate the effect of time (100 days) and treatment on genetic damage, and simple regression analysis to evaluate the relationship between exposure time to the substrate and genetic damage. The asterisks denote significant differences between treatments by exposure time with *p* < 0.05 (Tukey). ANOVA test: *** = *p* < 0.001, ** = *p* < 0.01, * = *p* < 0.05.

Our results show that there is a significant effect of time, treatment, and time×treatment interaction on the genetic damage in leaf tissue of *C. pumila* individuals (Figure 5).

In general, we observed a statistically significant increase in genetic damage levels over time in plants grown on both substrates, presenting significant differences between them for all exposure times. Individuals grown on mine trailing/biochar showed significantly lower levels of genetic damage compared to those grown on mine tailing (Figure 5).

In addition, we found a positive and significant relationship between exposure time and genetic damage for both treatments as the exposure time increased, higher levels of genetic damage (single-strand DNA breaks) were registered in *C. pumila* individuals (Figure 5).

## 3. Discussion

### 3.1. Influence of Biochar on Mine Tailing Substrate in the Bioaccumulation of Heavy Metals in C. pumila Individuals

*Crotalaria pumila* bioaccumulated HMs (Pb > Zn > Cu > Cd) during 100 days of exposure. In general, significant differences were found between treatments for all exposure times. The individuals growing on mine tailing with biochar were those that presented significantly lower levels of HM bioaccumulation compared to those growing on mine tailing. These results are similar to those found by Chen et al. [23], who carried out a meta-analysis of the effect of biochar on the availability of HM and its absorption by plants. They found that with high concentrations of biochar (≥5%), there was a reduction in metal bioaccumulation in plant tissues due to better HM retention and immobilization. In other words, the higher the concentration of biochar, the less concentration of HM in the substrate, reaching percentages of 38, 39, 35, and 17% for Cd, Pb, Cu, and Zn, respectively.

In this study, the *C. pumila* individuals growing on substrate contaminated with HM managed to significantly reduce the bioaccumulation of HM in both roots and leaves when the mine tailing was combined with biochar, made with coconut fiber. Hence, the addition of biochar is an effective technique that reduces the bioavailability of HMs. In addition, the use of biochar is recommended for stabilizing the soil against decomposition (it is recalcitrant) and retaining more nutrients in comparison with other forms of organic matter [24]. Furthermore, biochar made from organic waste reduces soil contamination and improves soil properties [24,25].

The coconut fiber biomass used in this study represents an important source of raw material for biochar production. Specifically, it is useful to stabilize HM in soils together with plant species capable of bioaccumulating HMs from soils. Furthermore, coconut fiber presents various advantages with respect to other biomasses. For example, it has been reported that biochar obtained from coconut shells results in greater toughness and resistance to degradation, similar to the biochar obtained from wood [26], due to its high content of lignin and cellulose [27]. Even though coconut shells are used for artisan purposes [27], their availability is high. There is no lack of this raw material, so it does not compete for use as a biochar precursor, which is a fundamental aspect when choosing the correct biomass to produce biochar [26]. Finally, coconut shells present a granular and porous structure, which enables them to retain water; in fact, they retain water up to nine times their weight [26]. This is an important feature when adding biochar to soil because in arid and semiarid zones, particularly in the dry season, plants do not have enough water, which results in poor development and a poor rate of survival. By adding biochar to the soil, its high capacity for absorbing water means it is able to store large quantities of water in the soil enabling plants to survive [28].

Various studies have reported similar results to those obtained in this study, where plant species that grow on HM contaminated soil, with added biochar, have resulted in a decrease in HM concentrations in their tissues [13,15,29,30,31,32,33].

Different procedures have been reported showing that when biochar interacts with HM, they are absorbed and immobilized, leading to a decrease in their bioavailability [34]. In this sense, Zhang et al. [34] and Xiao et al. [35] document that surface functional groups like hydroxyl, carboxyl, and phenol which contain oxygen, show a high capacity for sorption of inorganic contaminants like HM, where superficial complexation, ion exchange, and electrostatic attraction are the principal mechanisms [36].

Lu et al. [15] describe various routes whereby HM are absorbed by biochar, where the formation of precipitates such as phosphates is found, as well as increases in electrostatic attraction and ion exchange between the metallic cations and the biochar protons. In the same way, Puga et al. [13] explain that biochar interacts with HM through electrostatic attractions between metallic cations and the surface carbon with a negative charge, and ion exchange between ion protons on the surface carbon and the metallic cations. As mentioned above, the functional groups that contain oxygen in their structure are the main mechanisms for their absorption, with electrostatic attraction and ion exchange being the most frequently mentioned processes, resulting in biochar retaining and immobilizing HM.

Other studies report procedures where biochar immobilizes some HM; for example, Cu. Meier et al. [37] describe that functional groups, mainly hydroxyl, contained in biochar made with chicken manure, presented high affinity for Cu. Rechberger et al. [38] and Zhang et al. [39] mention that carbonates and hydroxides present in the ashes of biochar made with wood chips and bamboo, respectively, can be important absorbers of Cu (ll), since they are capable of promoting the formation of copper carbonate and copper hydroxide. As for Cd, Cheng et al. [40] mention that alkaline substances such as carbon trioxide, orthophosphate, and hydroxyl contained in biochar, have good capacities for adsorption and bonding to Cd, so this element transforms into copper hydroxide, copper phosphate, and copper carbonate [38].

As for Pb, Cheng et al. [40] mention that cationic exchange and precipitation reactions between basic substances, such as carbonates and hydroxides, also with Ca^2+^, Mg^2+^ present in biochar, are capable of immobilizing Pb, forming stable lead complexes. Wang et al. [41] and Ho et al. [42] report that biochar made with bird manure and from sewage sludge, respectively, contain abundant phosphate groups that can form insoluble compounds such as pyromorphite and hydroxypyromorphite, therefore capable of immobilizing Pb.

### 3.2. Influence of Biochar on Bioconcentration and Translocation of Heavy Metals in C. pumila Individuals

The BCF of *C. pumila* for all the bioaccumulated metals was >1 in the individuals growing on the mine tailing substrate and <1 in the individuals growing on the mine tailing/biochar substrate. Consequently, using plant species capable of accumulating HM in combination with organic compost such as biochar diminishes the mobility of HM, therefore reducing its bioavailability, so that plants associated with biochar bioaccumulate less HM in both structures, which in turn leads to a reduced biomagnification of HM along the food chain, lowering the ecological risks derived from HM exposure.

The translocation factor of *C. pumila* for all the metals in both treatments was <1, which indicates that this species bioaccumulated more HM in roots than in leaf tissue. Consequently, we can consider this particular species as a good phyto-stabilizer.

Similar results were reported by Ehab et al. [43], Eissa [44], and Li et al. [45], who found that biochar reduced both bioconcentration and translocation factors, compared to plants without the addition of biochar, reporting that this observation is the result of metal precipitation in root tissues.

### 3.3. Influence of Biochar on Mine Tailing Substrate on the Biomass of C. pumila

*C. pumila* individuals grown on mine tailing with biochar presented greater biomass values, compared to individuals grown without biochar. Similarly other studies support our results where biochar added to polluted soils with HM had a positive effect on the growth and biomass of various plants like *Sedum plumbizincicola*, *Canavalia ensiformis*, *Mucana aterrima*, *Nicotiana tabacum*, *Oryza sativa*, *Pisum sativum,* and *Capsicum annuam* [13,31,32,33,46], compared to plants grown on contaminated soil without biochar.

As shown in previous studies, when biochar is added to contaminated soils with HM, it exerts a positive effect on growth and plant biomass. Two processes can explain these observations, (1) biochar application adds nutrients to the soil, improving their availability and capacity for water retention [24,32,33,36,47]. López et al. [28] document that biochar can increase soil fertility due to its high content of carbonized organic matter that favors the absorption of nutrients, such as N, P, K y Ca.; (2) biochar decreases the bioavailability of HM since they form complexes with metallic ions on their surface, reducing the toxicity of HM in plants, leading to improved growth and development [13,48].

Both procedures are exemplified by Uchimiya et al. [29], who reported that biochar stabilized HM and promoted the liberation of nutrients such as Na, Ca, K, Mg, P, and S contained in both soil and biochar. So, biochar can reduce the bioavailability of HM as well as help plant development, increasing the bioavailability of nutrients. In addition, and in agreement with our results, biochar made from coconut fiber contains 10.5 mg kg^−1^ of phosphorus, which is found in the medium range in accordance with the norm NOM-021-SEMARNAT-2000. We therefore suggest that soils should be enriched with P for future absorption by plants.

### 3.4. Influence of Biochar on the Mine Tailing Substrate on Chlorophyll a and b Contents and Genetic Damage in C. pumila

It was found that the *C. pumila* individuals that grow on mine tailing with biochar had higher levels of chlorophyll *a* and *b* and lower levels of genetic damage, compared to individuals that grow on mine tailing with no biochar added.

Photosynthesis is a physiological process that plays a crucial role in the growth and development of plants [49]. Among the metals that have a negative influence on photosynthesis is lead, and our results show that Pb was the most bioaccumulated metal in *C. pumila*. Lead alters the absorption of essential nutrients such as Fe and Mg in plants. As a consequence, it inhibits the synthesis of chlorophyll *a* and *b* [20]. Also, in electron transport systems, it is known that Pb affects the donor and receiving sites of the photosystem II [50]. It is also known that HM, especially Pb, can substitute Mg ion in the chlorophyll molecule, which makes the capture of photons impossible, generating a decrease in the photosynthetic activity [20].

Cadmium can also negatively affect photosynthesis in low concentrations. It has been documented that Cd reduces growth, inhibits stomatic opening, chlorophyll synthesis, and photosynthesis, as well as causing chlorosis in the leaves, diminishing the concentration of carotenoids, and affecting the transpiration rate [20,51]. One of the most common symptoms of Cd toxicity is the chlorosis produced by an iron deficiency [52]. In addition, Cd causes imbalances in chloroplast metabolism, inhibiting chlorophyll synthesis and reducing the activity of enzymes implicated in CO_2_ fixation [53]. Kupper et al. [54] report that the stable bonding of Cd with proteins damages the photosynthetic apparatus, in particular photosystems I and II in *Thlaspi caerulencens*. Also, Cd can bond competitively with calcium essential binding sites in photosystem II during the photoactivation of the water division system, affecting photosynthesis [55].

Overall, we conclude that lead and cadmninum, which are non-essential and toxic in low concentrations, can negatively influence chlorophyll synthesis, which in turn affects photosynthesis, a fact that explains the results obtained in this study. However, we found that the individuals that grew on mine tailing substrate with biochar presented higher chlorophyll *a* and *b* contents, resulting from the reduction in HM bioaccumulation in these individuals, thus positively influencing the production of chlorophyll. Therefore, biochar plays a fundamental role in metal absorption, reducing their bioavailability, thereby decreasing toxic effects on photosynthesis.

In addition, we reported in this study that individuals growing on mine tailing substrate presented higher levels of genetic damage. Similar results have been reported in *Nicotiana tabacum* [56], *Prosopis laevigata* [57], and *Dodonaea viscosa* [58].

It is well known that HM can damage DNA in different ways, both directly and indirectly. Directly, HM can enter the cell nucleus and bind to the purine and pyrimidine bases, causing single- and double-stranded DNA breakage, leading to DNA damage [59]. Indirectly, it has been extensively reported that HM induces oxidative stress, favoring the production of Reactive Oxygen Species (ROS), increasing the level of free radicals in the cells, and negatively affecting cell macromolecules, as well as DNA [60]. In particular, Pb and Cd are high precursors of genetic damage when they are bioaccumulated in plants. In this sense, Pb indirectly induces single- and double-stranded DNA breakage. It can also replace Zn in the repair and replication enzymes with zinc fingers, as well as cause oxidative stress [61]. In addition, Pb is involved in the production of ROS [60] generating genetic damage [62]. Cd also produces oxidative stress, which promotes membrane and proteins alterations [63,64], as well as DNA base pair oxidations that lead to single- and double-stranded DNA breakage [52]. Since Pb was the most bioaccumulated metal in *C. pumila*, we suggest that it is the main responsible for the DNA damage observed in *C. pumila* leaf tissue.

## 4. Materials and Methods

### 4.1. Study Sites

This study was carried out in the mining district of Tlalquitenango, in the state of Morelos, Mexico. Metallic mining prevailed in the late 1990s, exploiting silver, lead, and zinc [23]. As a result of the extractive activities, 780,000 tons of mine tailings were deposited in two tailings (tailing 1: 18°26′07.7″ N–99°01′16.9″ W and tailing 2: 18°26′20.9″ N y 99°01′54.9″ W) at 500 and 1000 m from Huautla town, respectively. Both tailings have similar physicochemical characteristics: pH: 7.85 a 8.37, electric conductivity: 0.2 a 0.4 ds/m, percentage of organic matter: 0.52 a 0.84, particle size < 45 µm, and they are located at an altitude of 974 m [65,66]. HM concentrations (mg kg^−1^) were analyzed by atomic absorption spectrophotometry using the flame method (GBC 908 A, GBC Scientific Equipment Pty Ltd., Keysborough, Australia). HM concentrations in the tailing substrate are: Cu (8), Cd (8), Pb (46), and Zn (428) metal concentrations. The mining waste was randomly collected at both tailings sites. Each sample was obtained from the surface to a depth of 30 cm and mixed to obtain a homogeneous sample for both tailings.

### 4.2. Study Species

*Crotalaria pumila* Ortis (Fabaceae) is commonly known as “chipil”. *C. pumila* grows upright or upward, with stems between 30 and 50 cm long. Its floral phenology of fruit and seed maturation occurs from May to December. The fruit is an inflated legume 15 mm long by 8 mm in diameter, the seeds present physical latency [67]. *C. pumila* is a native species to Mesoamerica, which is distributed from South America to the southern United States of America. In Mexico, it is widely distributed, both in xerophilous scrubs, temperate forests, and dry tropical forest. *C. pumila* is a plant species that establishes, grows, and reproduces naturally in soils polluted with HMs. Finally, it is a species that has a primary use as food, and its cultivation is promoted for self-consumption [68,69].

### 4.3. Seed Collection, Germination, and Obtaining Seedlings

*Crotalaria pumila* seeds were collected from plants established in Quilamula, Tlaquiltenango, and Morelos (18°30′52″ N and 95°59′59″ W, in an altitude of 1070 m). In total, 20 plants were randomly sampled, from which 20% of the ripe fruits were collected [70]. For germination, the seeds were subjected to mechanical scarification due to their physical latency. Two hundred forty seeds were placed in Petri dishes (20 seeds per box) using filter paper as a substrate, which was moistened with distilled water once a day. After seed germination, the seedlings were placed in individual polyethylene bags (200 mL capacity) with Peat moss substrate until the formation of the first true leaves (15 days). Finally, a total of 180 individuals (90 plants per treatment, mine tailing/agrolite substrate (75:25, *v*/*v*), and mine tailing/biochar coconut fiber substrates [75:25, *v*/*v*]) were transplanted into individual nursery polyethylene bags (1 L capacity). Agrolite and biochar were sieved with a stainless-steel sieve with a mesh aperture of 0.5 mm (Fiicsa brand, Mexico City, Mexico), to obtain a substrate texture similar to the mine tailings (hereafter, mine tailing/agrolite substrate will be indicated as mine tailing substrate). All plants remained under greenhouse conditions, watered once a day, three times a week, at a temperature between 32 and 35 °C. Twelve individuals per treatment were randomly sampled. In each plant, HM bioaccumulation in roots and leaves, biomass, genetic damage, and chlorophyll content were measured. These evaluations were carried out every 25 days of exposure for both treatments until the 100 day period was accomplished.

### 4.4. Biochar Production and Physicochemical Characterization

Biochar was produced from coconut fiber from agro-industrial wastes at 600 °C through a pyrolysis process. For physicochemical characterization, biochar was considered as soil, pH, organic matter percentage, total carbon, and nitrogen determinations were carried out according to the Mexican standard [71].

### 4.5. Determination of Plant Biomass

To determine the effect of coconut fiber biochar on plant development, physiological and biomass characters were evaluated: fresh aerial part biomass, fresh root biomass, dry aerial part biomass, and dry root biomass.

### 4.6. Analysis of Heavy Metal Concentration

Three coconut fiber biochar samples were analyzed for metal content (Cd, Cu, Pb, and Zn). Twelve samples of the mine tailing substrate and mine tailing/biochar substrate were analyzed every 25 days to determine four metals (Cd, Cu, Pb, and Zn) in the root and leaf tissue of *C. pumila*. Root and leaf tissues were washed with running tap water and rinsed with distilled water three times to remove substrate residues. To ensure this removal, roots were observed under a stereoscopic microscope. Clean tissues were oven-dried to constant weight (60 °C, 72 h). Samples of 0.25 g of dry tissues of each structure were pulverized and poured in teflon bombs for acid digestion in a Microwave Accelerated Reaction System (CEM^®^ MARS-5); for acid digestion, a mixture of HNO_3_ (70%) and HCl (30%) was added to tissue samples. Samples were dissolved and filtered in distilled water to a final volume of 50 mL until analysis was performed. A non-tissue sample was simultaneously processed and used as a negative control. Finally, metal concentrations were analyzed by atomic absorption spectrophotometry using the flame method (GBC 908 A, GBC Scientific Equipment Pty Ltd., Keysborough, Australia). The concentration of Cd, Cu, Pb, and Zn was determined by calibration curves obtained using internal standard solutions of pure metal ions (ULTRA SCIENTIFIC). The standard calibration curves showed correlation coefficients (R^2^) between 0.995 and 1.0. The minimum detection limits (mg/L) according to the manufacturer for Cu (0.001), Cd (0.0004), Pb (0.001), and Zn (0.0005). The samples from the mine tailing substrate, mine tailing/biochar substrate, and coconut fiber biochar were processed simultaneously by triplicate. Average concentrations were reported in mg kg^−1^.

### 4.7. Determination of Bioconcentration Factor (BCF) and Translocation Factor (TF)

The heavy metal phytoextraction capacity of *C. pumila* was calculated using the bioconcentration factor (BCF) and the translocation factor (TF) indexes [72].

BCF_root_ = C[_root_]/C[_mine tailing_]

BCF_leaf_ = C[_leaf_]/C[_mine tailing_]

TF = C[_leaf_]/C[_root_]

where C[_mine tailing_] is the concentration in the mine tailing, and C[_leaf_] is the concentration of the metal detected in the leaf tissue, and C[_root_] is the concentration of the metal detected in the root tissue. It has been reported that if a plant presents values of TF > 1, as well as BCF > 1, the species is considered an accumulator for the analyzed metal [72,73].

A *t*-student test was performed to detect if there were significant differences between treatments (mine tailing and mine tailing/biochar substrate) in the bioconcentration factor (BCF) and the translocation factor (TF) for Cu, Cd, Pb, and Zn.

### 4.8. Genetic Damage: Alkaline Gel Electrophoresis

In total, 96 individuals were employed to determine genetic damage as single strand breaks, which was performed in 12 randomly chosen individuals for each exposure time (25, 50, 75, and 100 days) in both substrates (mine tailing and mine tailing/biochar). The alkaline single-cell gel electrophoresis assay, or “comet assay”, was performed as described by Tice et al. [72]. The selected leaves were washed with distilled water to remove impurities, and excess water was removed with absorbent paper towels. The leaf cuts were performed inside a glass Petri dish containing 200 µL of cold phosphate-buffered saline (PBS, pH 7.4) under dim light conditions. For nucleus isolation, cuts were made in the leaves with a sharp razor until the solution turned greenish; the plate was kept in ice for isolated nuclei collection. Subsequently, microscope slides were dipped in a 1% RMP (regular melting point) agarose aqueous solution (50 °C). After agarose was solidified, 50 μL of cell suspension was incorporated into an Eppendorf tube with 50 μL of low melting point agarose (1% LMPA), prepared on PBS, and the final mixture was poured over previously prepared slides. After that, a coverslip was placed over the mixture and preserved on ice. Once solidified, 80 µL of 0.5% LMP-agarose (37 °C) was added as a third layer, covered again with the coverslip, and left to solidify on ice (5 min). The coverslip was removed, and the slides with the isolated nuclei were immersed in cold lysis solution (100 mM Na_2_EDTA, 2.5 M NaCl, 10 mM fresh Tris, (pH 10), 1% Triton X100 and 10% dimethyl sulfoxide), and incubated at 4 °C for 24 h. After the lysis process, the slides were placed in electrophoresis tank with cold electrophoresis buffer (1 mM Na_2_EDTA and 300 mM NaOH, pH 13) and left there for 15 min for DNA unwinding. The electrophoresis was performed at 300 mA and 25 V for 20 min at 4 °C in the absence of light. After electrophoresis, the slides were neutralized (0.4 M Tris, pH 7.5) three times for 5 min and dehydrated with ethanol (100%). Finally, the slides were stained with 50 µL ethidium bromide (20 µg/mL), and samples were visualized at 400× under a fluorescent microscope (Carl Zeiss, Germany), 515–560 excitation filter, and a barrier filter of 590 nm. Fifty randomly selected nuclei were analyzed in each slide (100 nuclei per sample) using a computerized Image Analysis System (Comet Assay IV, Perceptive Instruments, Bury Saint Edmunds, UK). Tail length was used to evaluate the genetic damage levels caused by exposure to Cd, Cu, Pb, and Zn in mine tailing and mine tailing/biochar substrate.

### 4.9. Determination of Chlorophyll Content

The chlorophyll *a* and *b* content (mg/m^2^) were measured in 24 individuals (12 per substrate) for each exposure time (25, 50, 75, and 100 days) with a chlorophyll measuring device (ClorofiLOG, model CFL 1030). All readings were taken at the center of the leaf blade. The measurement was taken between 9:00 and 10:00 am. The chlorophyll measurements are taken in living tissues without causing damage, which allows the same leaf to be measured several times as it continues growing and developing. Measured values are expressed in dimensionless units, Falker Chlorophyll Index (FCI) values.

### 4.10. Statistical Analysis

The normality of the experimental data set was evaluated through the Shapiro–Wilk test (*W*) [73]. A two-factor analysis of variance was performed to evaluate the effect of the substrate (mine tailing and mine tailing/biochar), exposure time (25, 50, 75, and 100 days), and treatment per time interaction on biomass, chlorophyll content, and genetic damage in *C. pumila*. Subsequently, a post hoc (Tukey test, *p* < 0.05) was performed to establish significant differences in the average values of each analyzed character between substrates for each exposure time. Finally, simple regression analysis was performed to evaluate the relationship between exposure time to the substrate and each evaluated character [73].

Likewise, a two-factor analysis of variance was performed to determine the effect of the exposure time, treatment, and time × treatment interaction on the bioaccumulation of Cu, Cd, Pb, and Zn in the root and leaf of *C. pumila* individuals. Thereafter, a Tukey test was used to determine significant differences in the mean concentration of each analyzed metal between treatments for each exposure time [73].

The statistical analyses were carried out with the software STATISTICA version 8.0 [74].

## 5. Conclusions

The results of this study show that *C. pumila* can bioaccumulate high concentrations of non-essential metals such as Cd but manly Pb, as well as essential metals such as Cu and Zn, in root and leaf tissues. Hence, the present study shows that this species can be considered for phytoremediation purposes in polluted soils with HM. In addition, biochar made from coconut fiber is an efficient amendment for reducing the mobility of HM, decreasing its bioaccumulation and bioavailability, which positively influences the biomass and production of chlorophyll, as well as reducing the levels of genetic damage in the plant. In conclusion, these results suggest that *C. pumila* can be used in phytoremediation studies together with biochar made from coconut fiber, with the aim of: (a) reducing the bioaccumulation of HM in root and leaf tissue; (b) stabilizing HM in soils contaminated with Pb, Cu, Zn, and Cd; (c) increasing biomass and growth of *C. pumila* individuals, enabling a more effective phytoremediation strategy; (d) not affecting the production of chlorophyll; and (e) decreasing genetic damage in exposed plants. Finally, this system reduces the possibility of HM entering the food chain, thereby reducing the negative effects in ecosystem dynamics.

## Figures and Tables

**Table 1:** Heavy metal enrichment (BCF) and translocation values (TF) of Cu, Cd, Pb and Zn (Average ± standard deviation) in root and leaves of *C. pumila* plants growing under greenhouse conditions. The *t*-student analyses document significant differences between treatments by plant structure (in all cases the degrees of freedom were 95).

		Concentration (mg kg^−1^)					
Metals	Time (Days)	BCF (Root)		BCF (Leaf)		TF	TF
		Mine Tailing	Mine Tailing/Biochar	Mine Tailing	Mine Tailing/Biochar	Mine Tailing	Mine Tailing/Biochar
Zinc							
	25	1.15	0.37	0.18	0.14	0.16	0.37
	50	1.47	0.95	0.28	0.23	0.19	0.24
	75	1.63	0.85	0.54	0.21	0.33	0.25
	100	1.90	0.76	0.57	0.21	0.30	0.28
	Average ± SD	1.54 ± 0.32	0.73 ± 0.26	0.39 ± 0.19	0.20 ± 0.04	0.25 ± 0.08	0.29 ± 0.06
	*t*-student	24.049 ***		12.213 ***		3.917 ***	
Cooper							
	25	18.70	14.00	12.71	10.50	0.68	0.75
	50	26.67	18.00	14.71	11.88	0.55	0.66
	75	28.29	11.25	15.46	6.63	0.55	0.59
	100	26.30	9.25	13.84	5.13	0.53	0.55
	Average ± SD	24.99 ± 4.28	13.13 ± 3.79	14.18 ± 1.18	8.53 ± 3.18	0.58 ± 0.07	0.63 ± 0.09
	*t*-student	28.174 ***		22.237 ***		5.537 ***	
Lead							
	25	124.35	51.57	28.26	8.86	0.23	0.17
	50	157.20	59.71	58.09	11.71	0.37	0.20
	75	167.53	67.43	56.51	12.57	0.34	0.19
	100	140.28	44.00	55.22	8.14	0.39	0.19
	Average ± SD	147.34 ± 19	55.68 ± 10.13	49.52 ± 14.22	10.32 ± 2.15	0.33 ± 0.07	0.18 ± 0.01
	*t*-student	55.367 ***		34.707 ***		22.768 ***	
Cadmium							
	25	12.79	3.93	6.69	1.67	0.52	0.42
	50	15.90	3.57	6.10	1.31	0.38	0.37
	75	15.30	2.62	5.74	1.07	0.38	0.41
	100	14.58	1.79	3.23	0.00	0.22	0.00
	Average ± SD	14.64 ± 1.35	2.98 ± 0.97	5.44 ± 1.53	1.01 ± 0.12	0.38 ± 0.12	0.30 ± 0.20
	*t*-student	89.419 ***		33.574 ***		3.462 ***	

*** = *p* < 0.0001.

**Table 2:** Classification of *C. pumila* into Excluder, Indicator, Accumulator, and Hyperaccumulator of heavy metals (Cu, Cd, Pb, and Zn), according to Lam et al., 2023. Heavy metal concentrations in *C. pumila* were obtained after 100 exposure days.

					Predicted				
Type of Measurement	Metal	Sample []*_soil_*	Sample []*_plant_*	Adjusted BCF	Excluder	Indicator	Accumulator	Hyperaccumulator	Result
Root	Pb	6.972	910.27	370.39					Hyperaccumulator
	Cd	8.365	12.62	42.18	0.810	3.000	16.155	61.547	Accumulator
	Cu	8.023	211.75	74.76					Hyperaccumulator
	Zn	428.114	815.54	39.42	1.675	6.201	33.395	127.230	Accumulator
Leaves	Pb	6.972	132.3	145.81					Hyperaccumulator
	Cd	8.365	12.53	9.34	0.554	2.052	11.052	42.106	Accumulator
	Cu	8.023	111.05	39.21					Accumulator
	Zn	428.114	243.23	11.76	1.146	4.242	22.847	87.043	Accumulator

## Data Availability

Please contact author for data request.

## References

[1] Akoto R., Anning A.K. (2021). Heavy metal enrichment and potential ecological risks from different solid mine wastes at a mine site in Ghana. Environ. Adv..

[2] Mussali-Galante P., Tovar-Sánchez E., Valverde M., Rojas del Castillo E. (2013). Biomarkers of exposure for assessing environmental metal pollution: From molecules to ecosystems. Rev. Int. Contam. Ambient..

[3] Cuevas J.G., Faz A., Martínez S., Gabarrón M., Beltrá J.C., Martínez J., Acosta J.A. (2023). Spatial distribution and pollution evaluation in dry riverbeds affected by mine tailings. Environ. Geochem. Health.

[4] Raji I.B., Palamuleni L.G., Toxic Heavy Metals in Soil and Plants from a Gold Mining Area, South Africa (2023). Heavy Metals-Recent Advances. https://www.intechopen.com/books/12077#:~:text=10.5772/intechopen.104133.

[5] Chileshe M.N., Syampungani S., Festin E.S., Tigabu M., Daneshvar A., Odén P.C. (2023). Physico-chemical characteristics and heavy metal concentrations of copper mine wastes in Zambia: Implications for pollution risk and restoration. J. For. Res..

[6] Elhag M., Al-Ghamdi A.A.M., Galal H.K., Dahlan A. (2018). Evaluation of *Aloe vera* L. as phytoremediator of heavy metals contaminated soils in arid environments. Appl. Ecol. Environ. Res..

[7] Sharma J.K., Kumar N., Singh N.P., Santal A.R. (2023). Phytoremediation technologies and their mechanism for removal of heavy metal from contaminated soil: An approach for a sustainable environment. Front. Plant. Sci..

[8] Punia A. (2021). Role of temperature, wind, and precipitation in heavy metal contamination at copper mines: A review. Environ. Sci. Pollut. Res..

[9] Korzeniowska J., Stanislawska E. (2023). The Phytoremediation Potential of Local Wild Grass Versus Cultivated Grass Species for Zinc-Contaminated Soil. Agronomy.

[10] Bolan N.S., Park J.H., Robinson B., Naidu R., Huh K.Y. (2011). Phytostabilization: A green approach to contaminant containment. Adv. Agronomy.

[11] Singh H., Pant G. (2023). Phytoremediation: Low input-based ecological approach for sustainable environment. Appl. Water Sci..

[12] Zhang X., Wang H., He L., Lu K., Sarmah A., Li J., Huang H. (2013). Using biochar for remediation of soils contaminated with heavy metals and organic pollutants. Environ. Sci. Pollut. Res..

[13] Puga A.P., Abreu C.A., Melo L.C.A., Beesley L. (2015). Biochar application to a contaminated soil reduces the availability and plant uptake of zinc, lead and cadmium. J. Environ. Manag..

[14] Erakhrumen A.A., Agbontalor A. (2007). Phytoremediation: An Environmentally Sound Technology for Pollution Prevention, Control and Remediation in Developing Countries. Educ. Res. Rev..

[15] Lu K., Yang X., Gielen G., Bolan N., Ok Y.S., Niazi N.K., Wang H. (2017). Effect of bamboo and rice straw biochars on the mobility and redistribution of heavy metals (Cd, Cu, Pb and Zn) in contaminated soil. J. Environ. Manag..

[16] Ghorbani M., Konvalina P., Neugschwandtner R.W., Soja G., Bárta J., Chen W.H., Amirahmadi H.E. (2024). How do different feedstocks and pyrolysis conditions effectively change biochar modification scenarios? A critical analysis of engineered biochars under H_2_O_2_ oxidation. Energy Convers. Manag..

[17] FAO Coconut Food and Agricultural Organization of the United Nations. https://www.fao.org/faostat/en/#data/QCL.

[18] Ajien A., Idris J., Sofwan N., Husen R., Seli H. (2023). Coconut shell and husk biochar: A review of production and activation technology, economic, financial aspect and application. Waste Manag. Res..

[19] Rojas E., Lopez M.C., Valverde M. (1999). Single cell gel electrophoresis assay: Methodology and applications. J. Chromatogr..

[20] Pernía B., De Sousa A., Reyes R., Castrillo M. (2008). Biomarcadores de contaminación por cadmio en las plantas. Interciencia.

[21] Sánchez-López A.S., González-Chávez M.C.A., Carrillo-González R. (2017). Absorber, inmovilizar o atrapar: Funciones de las plantas en la remediación de sitios contaminados por elementos potencialmente tóxicos. Agro Product..

[22] Noguez-Inesta A., López-Sánchez A.S., Carrillo-González R., González-Chávez M.C.A. (2017). Uso de leguminosas (Fabaceae) en fitorremediación. Agro Product..

[23] Chen D., Liu X., Bian R., Cheng K., Zhang X., Zheng J., Li L. (2018). Effects of biochar on availability and plant uptake of heavy metals–A meta-analysis. J. Environ. Manag..

[24] Lehmann J. (2007). Bio-energy in the black. Front. Ecol. Environ..

[25] Escalante A., Pérez G., Hidalgo C., López J., Campo J., Valtierra E., Etchevers J.D. (2016). Biocarbón (biochar) I: Naturaleza, historia, fabricación y uso en el suelo. Terra Latinoam..

[26] Luna D., González A., Gordon M., Martín N. (2007). Obtención de carbón activado a partir de la cáscara de coco. ContactoS.

[27] Puentes L.H., Joya E. (2005). Reconocimiento de Características, Obtención y Utilización de la Estopa de Coco. Artesanías de Col..

[28] López M.I., Soledad B., Echezuría H., Delgado J. (2020). Evaluación de las características físicas del biocarbón obtenido por el Centro de Investigación y Desarrollo de Ingeniería de la UCAB. Tekhné.

[29] Uchimiya M., Chang S., Klasson K.T. (2011). Screening biochars for heavy metal retention in soil: Role of oxygen functional groups. J. Hazard. Mater..

[30] Melo L.C., Coscione A.R., Abreu C.A., Puga A.P., Camargo O.A. (2013). Influence of pyrolysis temperature on cadmium and zinc sorption capacity of sugar cane straw–derived biochar. BioResources.

[31] Shen X., Huang D.Y., Ren X.F., Zhu H.H., Wang S., Xu C., Zhu Q.H. (2016). Phytoavailability of Cd and Pb in crop straw biochar-amended soil is related to the heavy metal content of both biochar and soil. J. Environ. Manag..

[32] Zhang Y., Chen T., Liao Y., Reid B.J., Chi H., Hou Y., Cai C. (2016). Modest amendment of sewage sludge biochar to reduce the accumulation of cadmium into rice (*Oryza sativa* L.): A field study. Environ. Pollut..

[33] Nawab J., Ghani J., Khan S., Xiaoping W. (2018). Minimizing the risk to human health due to the ingestion of arsenic and toxic metals in vegetables by the application of biochar, farmyard manure and peat moss. J. Environ. Manag..

[34] Zhang C., Zeng G., Huang D., Lai C., Chen M., Cheng M., Wang R. (2019). Biochar for environmental management: Mitigating greenhouse gas emissions, contaminant treatment, and potential negative impacts. Chem. Eng. J..

[35] Xiao X., Chen B., Chen Z., Zhu L., Schnoor J.L. (2018). Insight into multiple and multilevel structures of biochars and their potential environmental applications: A critical review. Environ. Sci. Technol..

[36] Guo X.X., Liu H.T., Zhang J. (2020). The role of biochar in organic waste composting and soil improvement: A review. Waste Manag..

[37] Meier S., Curaqueo G., Khan N., Bolan N., Cea M., Eugenia G.M., Cornejo P., Ok Y., Borie F. (2017). Chicken-manure-derived biochar reduced bioavailability of copper in a contaminated soil. J. Soils Sediments.

[38] Rechberger M.V., Kloss S., Wang S.L., Lehmann J., Rennhofer H., Ottner F., Zehetner F. (2019). Enhanced Cu and Cd sorption after soil aging of woodchip-derived biochar: What were the driving factors?. Chemosphere.

[39] Zhang C., Shan B., Zhu Y., Tang W. (2018). Remediation effectiveness of Phyllostachys pubescens biochar in reducing the bioavailability and bioaccumulation of metals in sediments. Environ. Pollut..

[40] Cheng S., Chen T., Xu W., Huang J., Jiang S., Yan B. (2020). Application research of biochar for the remediation of soil heavy metals contamination: A review. Molecules.

[41] Wang Y., Lin Y., Chiu P.C., Imhoff P.T., Guo M. (2015). Phosphorus release behaviors of poultry litter biochar as a soil amendment. Sci. Total Environ..

[42] Ho S.H., Yang Z.K., Nagarajan D., Chang J.S., Ren N.Q. (2017). High-efficiency removal of lead from wastewater by biochar derived from anaerobic digestion sludge. Bioresour. Technol..

[43] Ehab I.A., El-Sherbini M.A., Selim E.M.M. (2022). Effects of biochar on soil properties, heavy metal availability and uptake, and growth of summer squash grown in metal-contaminated soil. Sci. Hortic..

[44] Eissa M.A. (2019). Effect of cow manure biochar on heavy metals uptake and translocation by zucchini (*Cucurbita pepo* L.). Arabian J. Geosci..

[45] Li H., Liu Y., Chen Y., Wang S., Wang M., Xie T., Wang G. (2016). Biochar amendment immobilizes lead in rice paddy soils and reduces its phytoavailability. Sci. Rep..

[46] Lu H., Li Z., Fu S., Mendez A., Gasco G., Paz J. (2014). Can biochar and phytoextractors be jointly used for cadmium remediation?. PLoS ONE.

[47] Smider B., Singh B. (2014). Agronomic performance of a high ash biochar in two contrasting soils. Agric. Ecosyst. Environ..

[48] Lebrun M., Macri C., Miard F., Hattab N., Motelica M., Morabito D., Bourgerie S. (2017). Effect of biochar amendments on As and Pb mobility and phytoavailability in contaminated mine technosols phytoremediated by Salix. J. Geochem. Explor..

[49] Taiz L., Zeiger E. (2015). Plant Physiology and Development.

[50] Rashid A., Camm E.L., Ekramoddoullah A.K. (1994). Molecular mechanism of action of Pb^2+^ and Zn^2+^ on water oxidizing complex of photosystem II. FEBS Lett..

[51] Sandalio L.M., Dalurzo H.C., Gomez M., Romero-Puertas M.C., Del Rio L.A. (2001). Cadmium-induced changes in the growth and oxidative metabolism of pea plants. J. Exp. Bot..

[52] Benavides M.P., Gallego S.M., Tomaro M.L. (2005). Cadmium toxicity in plants. Braz. J. Plant Physiol..

[53] Ali G., Srivastava P.S., Iqbal M. (2000). Influence of cadmium and zinc on growth and photosynthesis of *Bacopa monniera* cultivated in vitro. Biol. Plant.

[54] Küpper H., Parameswaran A., Leitenmaier B., Trtílek M., Šetlík I. (2007). Cadmium-induced inhibition of photosynthesis and long-term acclimation to cadmium stress in the hyperaccumulator *Thlaspi caerulescens*. New Phytol..

[55] Faller P., Kienzler K., Krieger A. (2005). Mechanism of Cd^2+^ toxicity: Cd^2+^ inhibits photoactivation of Photosystem II by competitive binding to the essential Ca^2+^ site. Biochim. Biophys. Acta Bioenerg..

[56] Gichner T., Patková Z., Száková J., Demnerová K. (2006). Toxicity and DNA damage in tobacco and potato plants growing on soil polluted with heavy metals. Ecotox. Environ. Saf..

[57] Muro-González D.A., Mussali-Galante P., Valencia-Cuevas L., Flores-Trujillo K., Tovar-Sánchez E. (2020). Morphological, physiological, and genotoxic effects of heavy metal bioaccumulation in *Prosopis laevigata* reveal its potential for phytoremediation. Environ. Sci. Pollut. Res..

[58] Castañeda-Espinoza J., Salinas-Sánchez D.O., Mussali-Galante P., Castrejón-Godínez M.L., Rodríguez A., González-Cortazar M., Zamilpa A., Tovar-Sánchez E. (2022). *Dodonaea viscosa* (Sapindaceae) as a phytoremediator for soils contaminated by heavy metals in abandoned mines. Environ. Sci. Pollut. Res..

[59] Siddiqui S., Meghvansi M.K., Wani M.A., Jabee F. (2009). Evaluating cadmium toxicity in the root meristem of *Pisum sativum* L.. Acta Physiol. Plant..

[60] Huihui Z., Xin L., Zisong X., Yue W., Zhiyuan T., Meijun A., Guangyu S. (2020). Toxic effects of heavy metals Pb and Cd on mulberry (*Morus alba* L.) seedling leaves: Photosynthetic function and reactive oxygen species (ROS) metabolism responses. Ecotoxicol. Environ. Saf..

[61] Pourrut B., Shahid M., Douay F., Dumat C., Pinelli E. (2013). Molecular mechanisms involved in lead uptake, toxicity and detoxification in higher plants. Heavy Metal Stress in Plants.

[62] Ercal N., Gurer-Orhan H., Aykin-Burns N. (2001). Toxic metals and oxidative stress part I: Mechanisms involved in metal-induced oxidative damage. Curr. Top. Med. Chem..

[63] Wu F., Zhang G., Dominy P. (2003). Four barley genotypes respond differently to cadmium: Lipid peroxidation and activities of antioxidant capacity. Environ. Exp. Bot..

[64] Balestrasse K.B., Gallego S.M., Tomaro M.L. (2004). Cadmium-induced senescence in nodules of soybean (*Glycine max* L.) plants. Plant Soil.

[65] SEMARNAT (2005). Evaluación de Tecnologías de Remediación Para Suelos Contaminados Con Metales, Etapa II.

[66] Mussali-Galante P., Santoyo-Martínez M., Castrejón-Godínez M.L., Breton-Deval L., Rodríguez-Solís A., Valencia-Cuevas L., Tovar-Sánchez E. (2023). The bioaccumulation potential of heavy metals by *Gliricidia sepium* (Fabaceae) in mine tailings. Environ. Sci. Pollut. Res..

[67] Rzedowski G.C., Rzedowski J. (2001). Flora fanerogámica del Valle de México.

[68] Lindig R., Lara S. (2004). Effect of scarification and growing media on seed germination of *Crotalaria pumila* (Ort.). Seed Sci. Technol..

[69] Sourakov A. (2015). You are what you eat: Native versus exotic *Crotalaria* species (Fabaceae) as host plants of the Ornate Bella Moth, *Utetheisa ornatrix* (Lepidoptera: Erebidae: Arctiinae). J. Nat. Hist..

[70] Gold K., León P., Way M. (2004). Manual de Recolección de Semillas de Plantas Silvestres.

[71] Mexicana, Norma Oficial (2000). NOM-021-RECNAT-2000. Establece las especificaciones de fertilidad, salinidad y clasificación de suelos. Estudios, muestreos y análisis. D. Of. Fed..

[72] Tice R.R., Agurell E., Anderson D., Burlinson B., Hartmann A., Kobayashi H., Sasaki Y.F. (2000). Single cell gel/comet assay: Guidelines for in vitro and in vivo genetic toxicology testing. Environ. Mol. Mutagen..

[73] Zar J.H. (2010). Biostatistical Analysis.

[74] Statsoft, Inc. (2007). Statistica for Windows.

